# Supplementary material for: Contributions of distinct gold species to catalytic reactivity for carbon monoxide oxidation
Source: Nat Commun. 2016 Nov 16;7:13481. doi: 10.1038/ncomms13481 (PMC5116099; doi:10.1038/ncomms13481)
Supplement: Supplementary Information — Supplementary Figures 1-12, Supplementary Table 1 and Supplementary Methods. [file ncomms13481-s1.pdf]

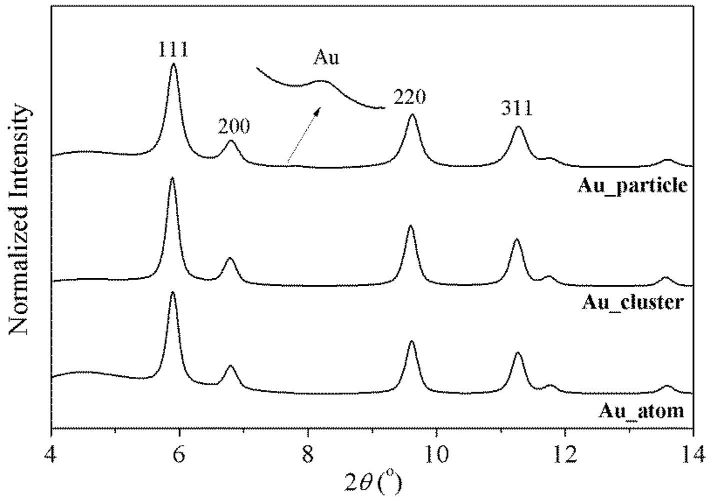

Supplementary Figure 1 | Result from XRD measurements. Synchrotron radiation XRD patterns of the as-prepared gold-ceria samples. The detailed information on XRD measurement is seen in the **Supplementary Methods**.

These data were collected under the *in-situ* experiments before the introduction of CO and O_2 reactants. Fluorite cubic CeO_2 ($F_{m\text{-}3m}$, JCPDS card #: 34-394) crystal structure was determined for all the measured samples. Besides, a minor phase of metallic Au ($F_{m\text{-}3m}$, JCPDS card #: 4-784) was detected for Au_particle only.

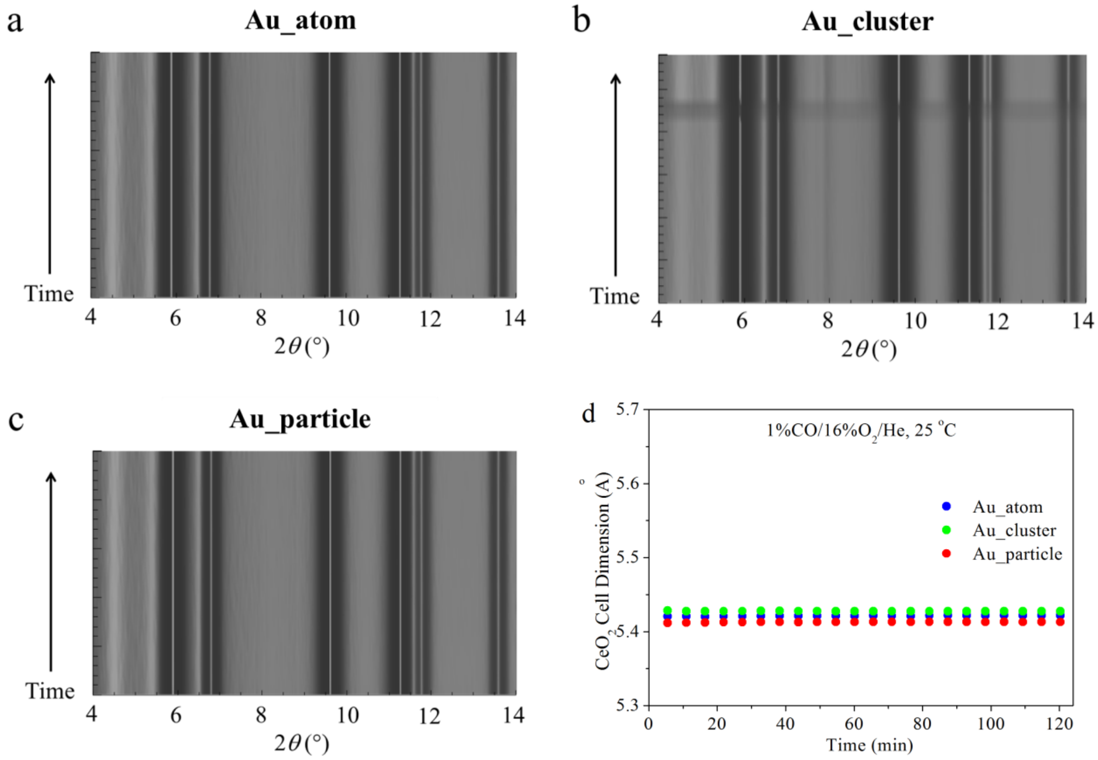

Supplementary Figure 2 | Results from *in situ* XRD measurements. Synchrotron radiation *in situ* XRD patterns of the gold-ceria samples: (**a**) Au_atom; (**b**) Au_cluster; (**c**) Au_particle; (**d**) CeO_2 cell dimension as a function of reaction time under the CO oxidation conditions (1%CO/16%O_2/He, 5 mL·min^{-1}, at 25 °C). The detailed information on *in situ* XRD measurement is seen in the **Supplementary Methods**.

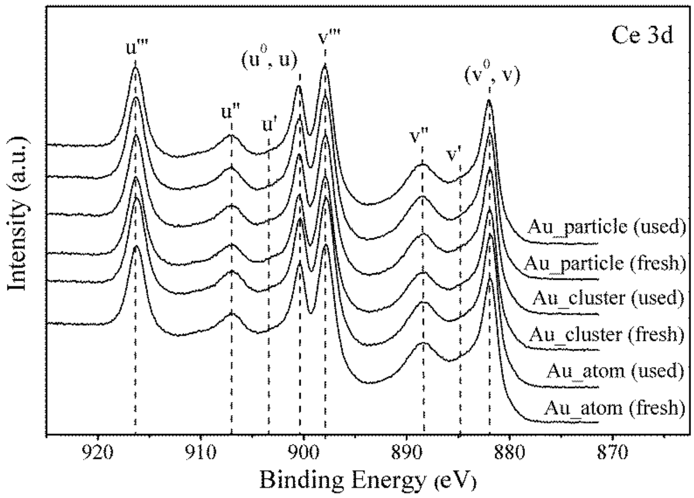

Supplementary Figure 3 | Results from XPS measurements. Ce 3d XPS spectra of different gold-ceria samples before and after the CO oxidation reaction. The detailed information on XPS measurement is seen in the **Supplementary Methods**.

The analysis on specific peaks in each Ce 3d spectrum to obtain the relative ratio of Ce^{3+}/Ce^{4+} was carried out accordingly to this equation:

$$Ce^{3+}/Ce^{4+} = Sv'/(Sv''+S(v^0,v)) \quad (1)$$

where Sv', Sv'' and $S(v^0,v)$ stand for the peak areas of v', v'' and (v^0,v), respectively.

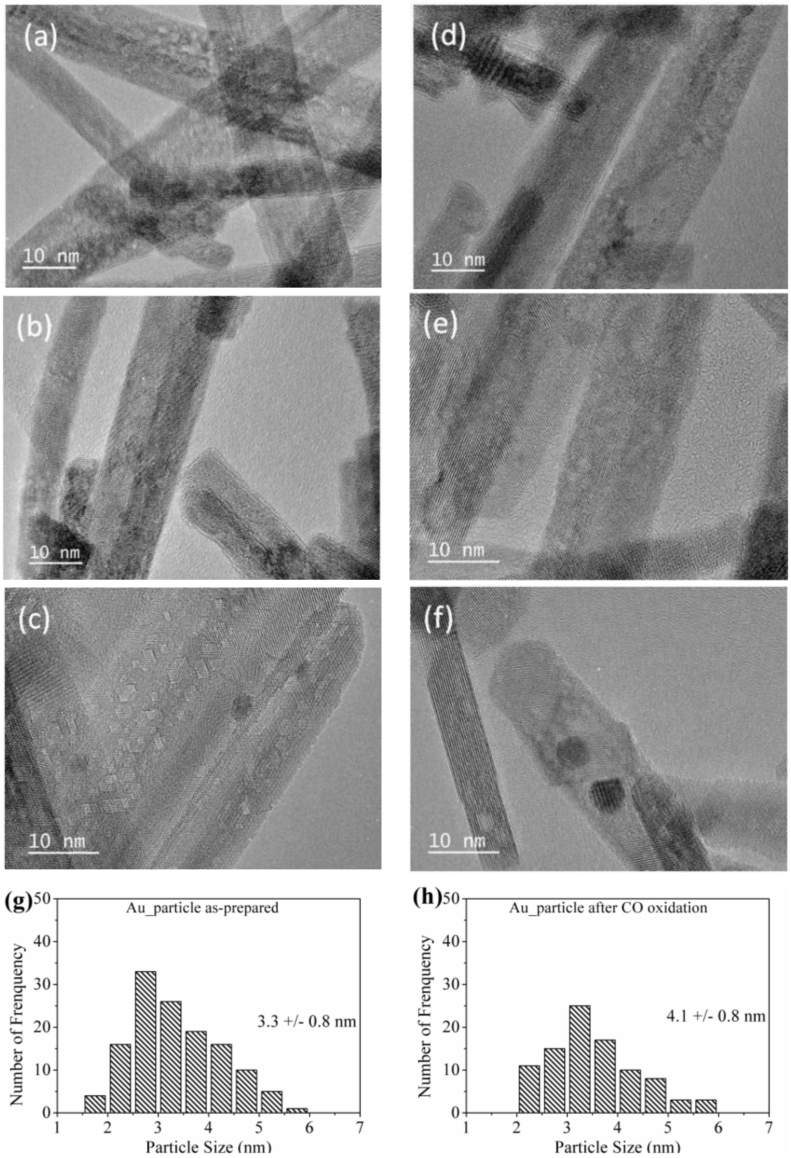

Supplementary Figure 4 | Results from HRTEM measurements. HRTEM images (**a-f**) and size-distribution histograms (**g**, **h**) of gold-ceria samples: (**a**) Au_atom, as-prepared; (**b**) Au_cluster, as-prepared; (**c**, **g**) Au_particle, as-prepared; (**d**) Au_atom, after CO oxidation; (**e**) Au_cluster, after CO oxidation; (**f**, **h**) Au_particle, after CO oxidation.

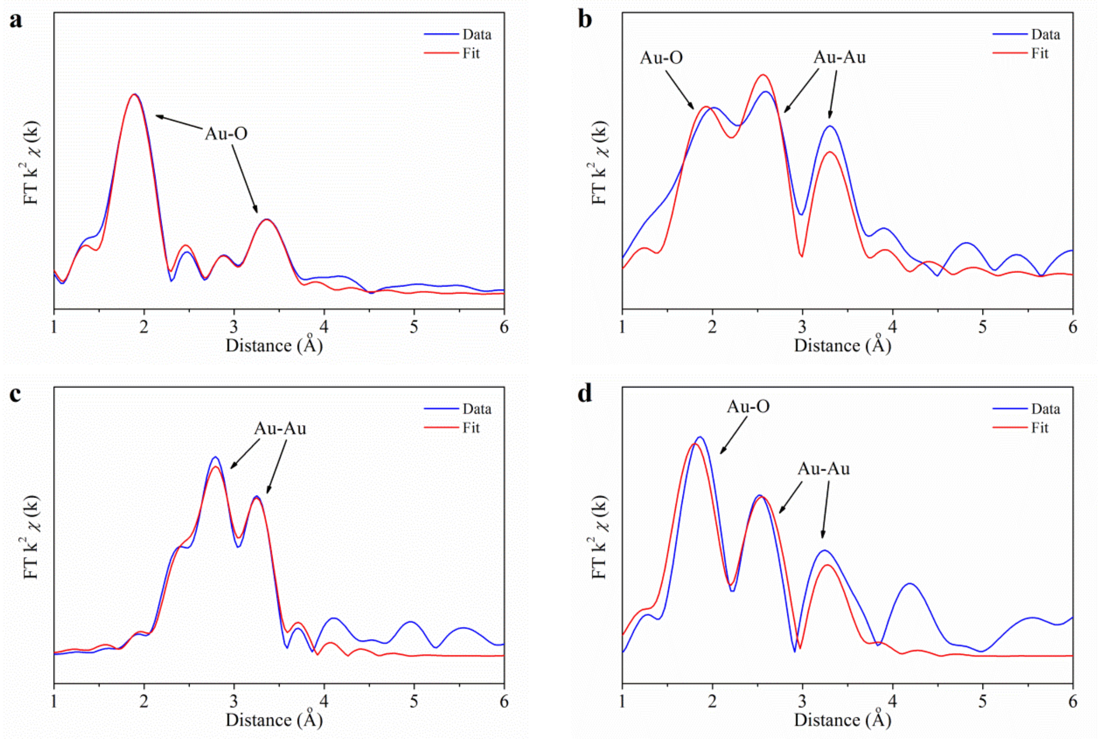

Supplementary Figure 5 | Fitting results from EXAFS measurements. EXAFS fitting results on the gold-ceria samples: (**a**) Au_atom, as-prepared; (**b**) Au_cluster, as-prepared; (**c**) Au_particle, as-prepared; (**d**) Au_cluster, *in situ*.

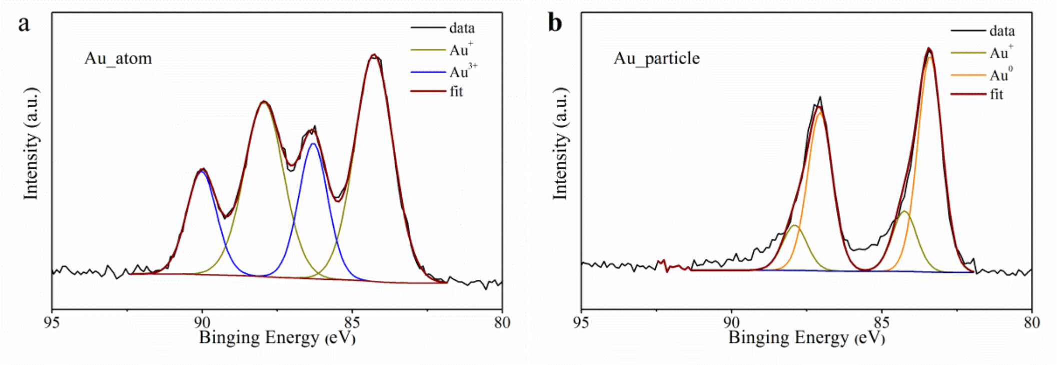

Supplementary Figure 6 | Results from XPS measurements. XPS peak deconvolutions on the references for XANES linear combination analysis: (**a**) Au_atom, as-prepared (Au^0:Au^+:Au^{3+}=0:69:31); (**b**) Au_particle, as-prepared (Au^0:Au^+:Au^{3+}=76:24:0). The detailed information on XPS measurement is seen in the **Supplementary Methods**.

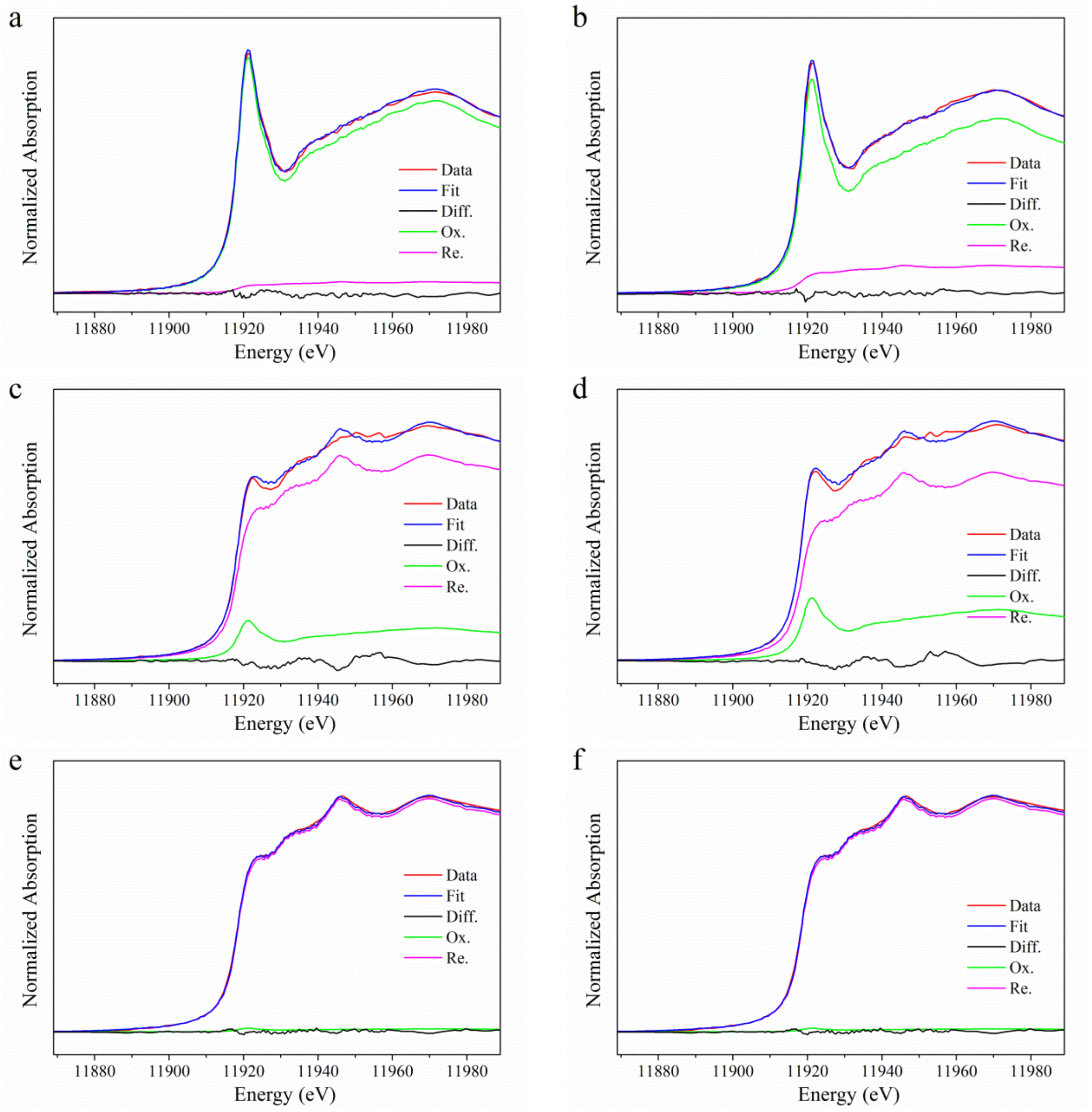

Supplementary Figure 7 | XANES results of the gold-ceria samples. Linear combination details on the XANES spectra of the gold-ceria samples at the start (**a**,**c**,**e**) and end (**b**,**d**,**f**) of the *in situ* measurements: (**a**,**b**) Au_atom; (**c**,**d**) Au_cluster; (**e**,**f**) Au_particle.

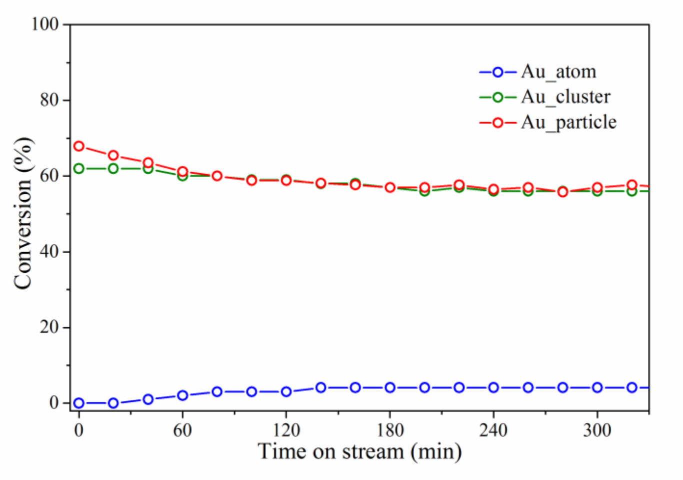

Supplementary Figure 8 | CO conversions of gold-ceria catalysts. CO conversions of gold-ceria catalysts under a steady-state condition (25 mg catalyst, 1%CO/20%O_2/N_2, 20 mL·min^{-1}, at 25 °C).

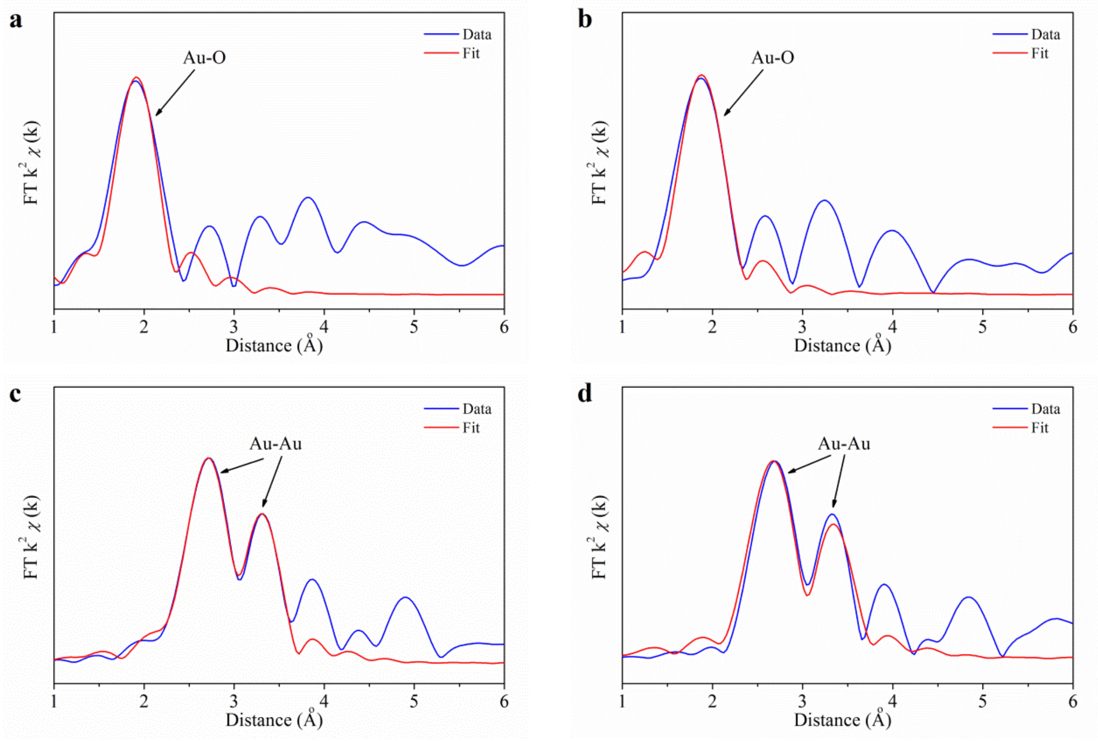

Supplementary Figure 9 | EXAFS fitting results of gold-ceria samples. EXAFS fitting results in *R* space of the gold-ceria samples at the start (**a**,**c**) and end (**b**,**d**) of the *in situ* measurements: (**a**,**b**) Au_atom; (**c**,**d**) Au_particle.

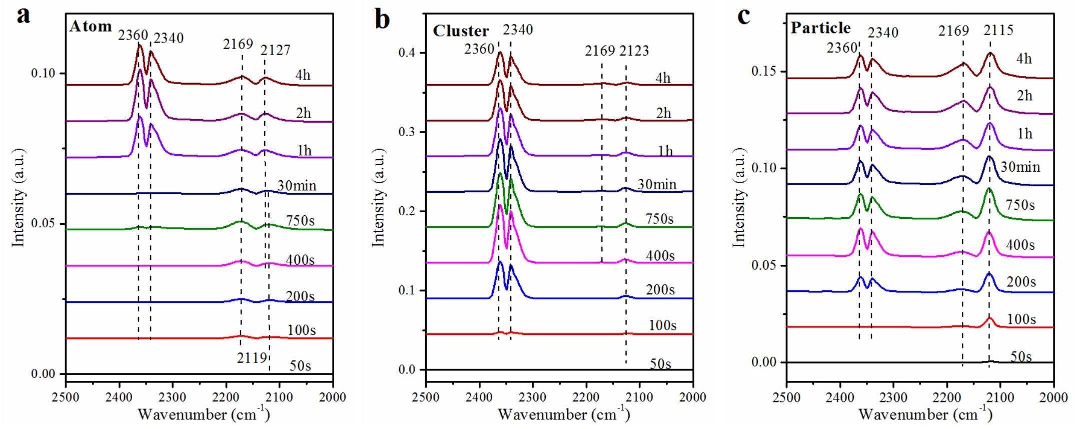

Supplementary Figure 10 | *In situ* DRIFTS results of gold-ceria samples. *In situ* DRIFTS spectra over gold-ceria samples of (**a**) Au_atom, (**b**) Au_cluster and (**c**) Au_particle under the CO oxidation reaction conditions (at 25 °C, 30 mg catalyst, 1%CO/20%O_2/N_2, 30 mL·min^{-1}).

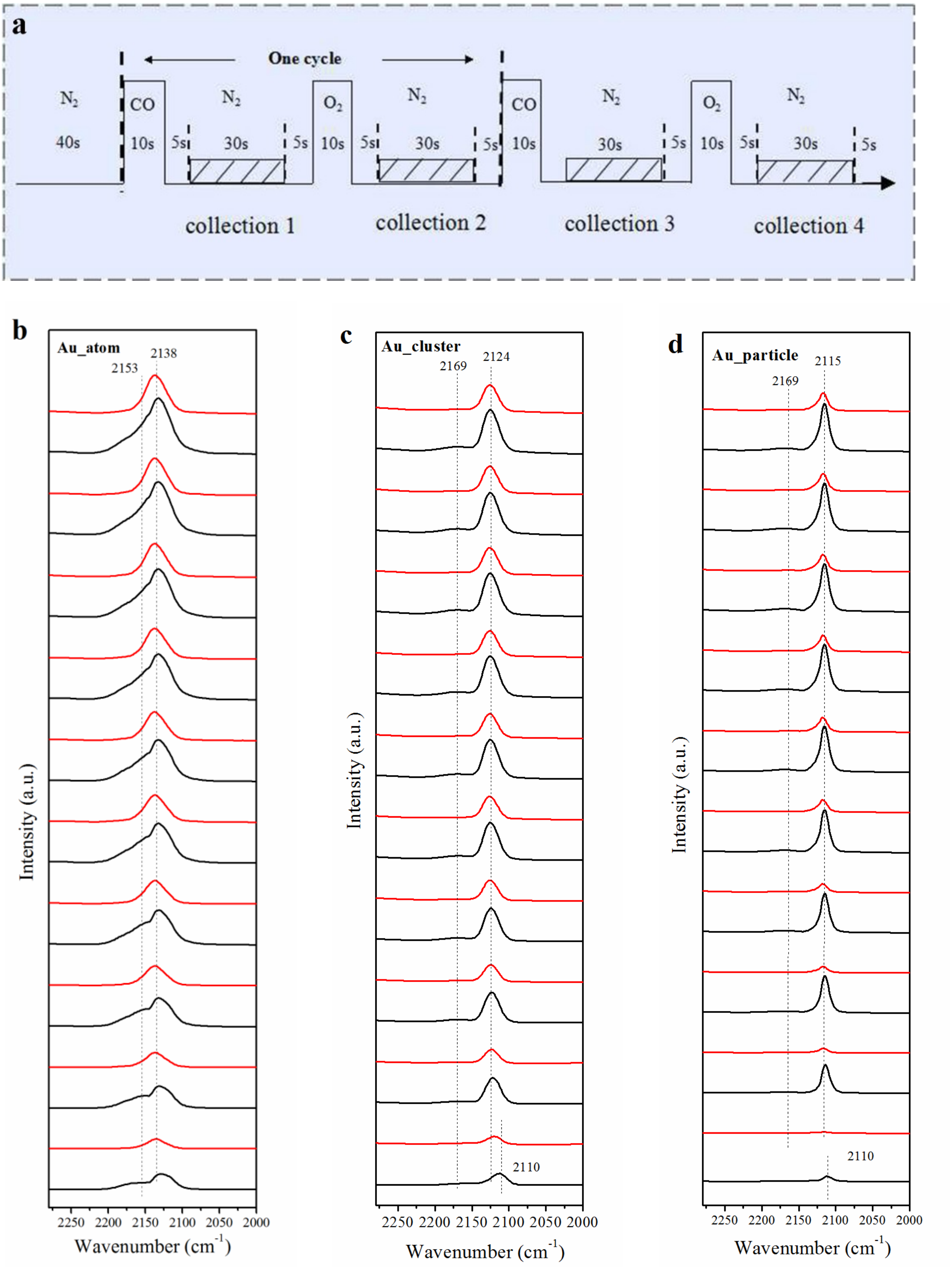

Supplementary Figure 11 | Pulsed adsorption-desorption DRIFT experiments results of gold-ceria samples. Schematic illustration on the pulsed adsorption-desorption experiments **(a)** and *in situ* DRIFT spectra collected from the first 10 circles (1–20 switches) in the pulsed adsorption-desorption experiments on gold-ceria samples: (**b**) Au_atom, (**c**) Au_cluster, (**d**) Au_particle (black: after CO adsorption; red: after O_2 adsorption).

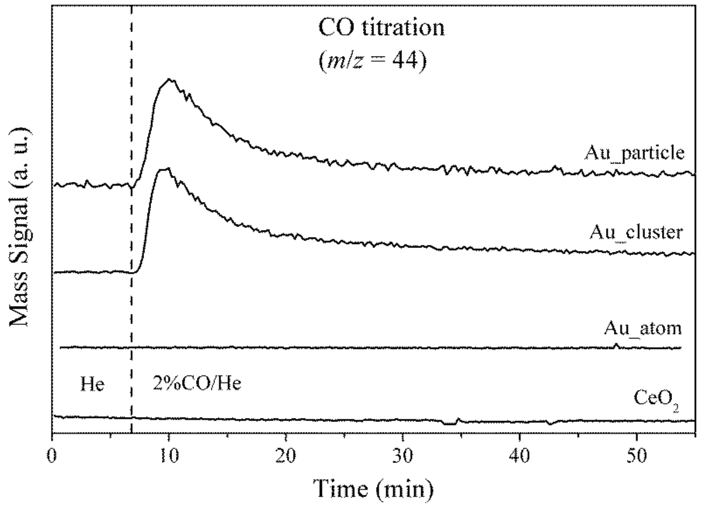

Supplementary Figure 12 | Results from CO titration. CO_2 (*m*/*z* = 44) evolution collected during CO titration over gold-ceria samples at room temperature. The detailed information on the CO titration tests is seen in the **Supplementary Methods**.

Supplementary Table 1 | Fitting results from EXAFS measurements. EXAFS fitting results on bond distance (*R*) and coordination number (CN) of Au-O and Au-Au shells for the gold-ceria samples during CO oxidation.

Sample	Au-O		Au-Au	
	R **(Å)**	**CN**	***R*** **(Å)**	**CN**
Au_atom_23min	1.98±0.01	3.4±0.6	–	–
Au_atom_44min	1.97±0.01	3.1±0.6	–	–
Au_atom_125min	1.97±0.02	3.5±0.7	–	–
Au_atom_165min	1.97±0.03	2.9±0.8	–	–
Au_atom_186min	1.98±0.02	3.0±0.6	–	–
Au_atom_226min	1.96±0.03	2.8±0.8	–	–
Au_atom_247min	1.97±0.02	3.4±0.7	–	–
Au_cluster[a]	1.94±0.02	0.9±0.3	2.80±0.02	4.7±1.5
Au_particle_30min	–	–	2.85±0.01	10.6±0.9
Au_particle_56min	–	–	2.85±0.01	8.5±0.9
Au_particle_109min	–	–	2.83±0.01	7.8±0.8
Au_particle_162min	–	–	2.84±0.01	9.0±1.1
Au_particle_189min	–	–	2.85±0.01	8.6±0.9
Au_particle_295min	–	–	2.85±0.01	8.8±1.0

[a]: For Au_cluster, the signal-to-noise level was not good enough to run the EXAFS fitting on each step of experiment, and thus averaged for all the *in situ* spectra.

Supplementary Methods

X-ray diffraction (XRD): The *ex situ* and *in situ* XRD experiments were carried out on X7B beamline (λ = 0.3196 Å) of NSLS at BNL. The powder sample (1–2 mg) was loaded into a quartz tube (I.D. = 0.9 mm, O.D. = 1.0 mm) which was attached to the same flow cell as in the XAFS measurements. One small resistance heating wire was installed right below the tube, and the temperature was monitored with a 0.5-mm chromel-alumel thermocouple that was placed inside the tube near the sample. The in-situ CO oxidation reaction was conducted under these conditions: 1%CO/16%O_2/He, 5 mL·min^{-1}, at 25 °C. Before each reaction, the gold-ceria sample was pretreated in either 20%O_2/He (300 °C, 30 min) for Au_atom/Au_particle or 5%H_2/He (300 °C, 30 min) for Au_cluster. Two-dimensional (2D) XRD patterns were collected with an image-plate detector (Perkin-Elmer), and the powder rings were integrated using the FIT2D code. The cell dimensions of CeO_2 were determined by Rietveld refinement.

X-ray photoelectron spectroscopy (XPS): The XPS analysis was carried out on an Axis Ultra XPS spectrometer (Kratos, U.K.) with Al Kα radiation operated at 225 W with 15 kV acceleration voltage. The peak at 284.8 eV of the C *1s* spectra was used to calibrate the binding energy. The analysis on specific peaks in each Ce *3d* spectrum to obtain the relative ratio of Ce^{3+}/Ce^{4+} was carried out accordingly to this equation: $Ce^{3+}/Ce^{4+} = Sv'/(Sv''+S(v^0,v))$, where Sv', Sv'' and $S(v^0,v)$ stand for the peak area of v', v'' and (v^0,v), respectively.

CO titration tests: The CO titration tests were performed at Builder PCSA-1000 System equipped with a mass spectrometer (AMETECK, DYCOR LC-D200). First, ca. 100 mg sample powders were pretreated at 300 °C (10 °C/min) in air (50mL/min) for 30 min and in addition subsequent pretreatment (300 °C, 5%H_2/He, 30 min) was required for Au_cluster. After cooling down, the measured sample was continued to be purged with pure O_2 (50 mL/min) at room temperature for 1 h. Then, the feed gas was switched to pure He (30 mL/min) at room temperature until the stabilization of baseline. The CO titration process was done in 2%CO/He (30 mL/min) at room temperature with the simultaneous collection on CO_2 signals (m/z = 44).
